# Functional and Transcriptomic Characterization of Peritoneal Immune-Modulation by Addition of Alanyl-Glutamine to Dialysis Fluid

**DOI:** 10.1038/s41598-017-05872-2

**Published:** 2017-07-24

**Authors:** Rebecca Herzog, Lilian Kuster, Julia Becker, Tobias Gluexam, Dietmar Pils, Andreas Spittler, Manoj K. Bhasin, Seth L. Alper, Andreas Vychytil, Christoph Aufricht, Klaus Kratochwill

**Affiliations:** 1 Medical University of Vienna, Department of Pediatrics and Adolescent Medicine, Vienna, Austria; 2 Medical University of Vienna, Christian Doppler Laboratory for Molecular Stress Research in Peritoneal Dialysis, Vienna, Austria; 3 Medical University of Vienna, Center for Medical Statistics, Informatics, and Intelligent Systems, Vienna, Austria; 4 Medical University of Vienna, Department of Surgery, Research Laboratories & Core Facility Flow Cytometry, Vienna, Austria; 5 Division of Interdisciplinary Medicine, Beth Israel Deaconess Medical Center, Boston, MA USA; 6 Department of Medicine, Harvard Medical School, Boston, MA USA; 7 Division of Nephrology and Center for Vascular Biology Research, Beth Israel Deaconess Medical Center, Boston, MA USA; 8 Medical University of Vienna, Department of Medicine III, Division of Nephrology and Dialysis, Vienna, Austria

## Abstract

Peritonitis remains a major cause of morbidity and mortality during chronic peritoneal dialysis (PD). Glucose-based PD fluids reduce immunological defenses in the peritoneal cavity. Low concentrations of peritoneal extracellular glutamine during PD may contribute to this immune deficit. For these reasons we have developed a clinical assay to measure the function of the immune-competent cells in PD effluent from PD patients. We then applied this assay to test the impact on peritoneal immune-competence of PD fluid supplementation with alanyl-glutamine (AlaGln) in 6 patients in an open-label, randomized, crossover pilot trial (EudraCT 2012-004004-36), and related the functional results to transcriptome changes in PD effluent cells. *Ex-vivo* stimulation of PD effluent peritoneal cells increased release of interleukin (IL) 6 and tumor necrosis factor (TNF) α. Both IL-6 and TNF-α were lower at 1 h than at 4 h of the peritoneal equilibration test but the reductions in cytokine release were attenuated in AlaGln-supplemented samples. AlaGln-supplemented samples exhibited priming of IL-6-related pathways and downregulation of TNF-α upstream elements. Results from measurement of cytokine release and transcriptome analysis in this pilot clinical study support the conclusion that suppression of PD effluent cell immune function in human subjects by standard PD fluid is attenuated by AlaGln supplementation.

## Introduction

Infectious complications of peritoneal dialysis (PD) remain the major causes for therapeutic failure^1–3^. Peritonitis, in particular, is one of the main predictors of long-term morbidity and mortality in PD patients^1, 4^. Several studies have shown currently available PD fluids (PDFs) to be bioincompatible due to acidity, buffer composition, high glucose content, hyperosmolarity, and presence of glucose degradation products^5–8^. Analyses of effects of PDF on peritoneal leukocyte function demonstrate reduced peritoneal immune-competence, potentially contributing to increased risk of PD-related peritonitis^9–12^. However, deciphering the complex interplay of inflammation, stress responses, and host defence requires application of both functional assays and powerful global analytic methods.

The introduction of multi-chamber bags in PD has increased preclinical biocompatibility of PDF by allowing more physiologic fluid compositions and better sterilization procedures than achievable with single-chamber bags^13^. However, a recent Cochrane meta-analysis on the use of biocompatible PDFs concluded that the rate of infectious complications was not significantly improved by these measures^14^. These data emphasize the ongoing medical need to understand and prevent infectious complications of PD.

The prohibitive costs of clinical PD trials based on hard outcomes such as peritonitis require development of reliable surrogate outcome parameters for early clinical development of improved formulations of PDFs. In previous studies focusing on different aspects of systemic immune-competence, whole-blood culture assays were developed and cytokine content was analyzed in diluted heparinized blood after *ex-vivo* exposure to toll-like receptor (TLR) agonists^15^. This method as reported was sufficiently reproducible to detect meaningful changes in clinical trials of relatively small sample size^15^. In the intensive care setting, *ex-vivo* cytokine release has been shown to correlate with clinical outcomes, establishing this functional assay as a useful biomarker for tests of novel therapeutic interventions^16–18^.

Interestingly, addition of glutamine results in improved post-operative *ex-vivo* cytokine release^19^. A Cochrane meta-analysis on glutamine (Gln) supplementation of parenteral nutrition in critically ill patients demonstrated that systemic Gln administration reduced infection rate in this vulnerable patient population^20^. The use of comparable tools in clinical PD research, however, has not attained broad acceptance, likely reflecting high complexity and low practicability of the surrogate assays tested to date^6^.

Understanding the underlying molecular biological connections of such a test is of critical importance. Omics technologies represent an attractive approach for gaining insight into the complex mechanisms of PD-induced immune-suppression and its potential pathobiological effects on clinical outcomes. Transcriptomics techniques are currently the most widely used of these tools, with microarray-based techniques measuring normalized nucleotide sequence abundance values. In contrast, RNA sequencing (RNA-seq) overcomes the limitations of genetic variation of patients and the complexity of transcriptional regulation by alternative splicing^21^. Thereby RNA-seq allows de-novo analysis of miniscule amounts of cells as expected in PD effluents without prior definition of a consensus genome or transcripts of interest.

In this study we test the feasibility of a simple method for monitoring function of peritoneal immune-competent cells in clinical practice, based on an adaptation of the *ex-vivo* lipopolysaccharide (LPS)-stimulated cytokine release assay^6, 22, 23^. We next use this streamlined assay in a randomized pilot-feasibility trial to assess effects of a novel PDF supplemented with the stable Gln dipeptide alanyl-glutamine (AlaGln). We then employ a multi-level transcriptomic approach to gain insight into the regulatory mechanisms leading to increased peritoneal immune-competence. The resultant increased understanding of potential cytoprotection targets should facilitate translation of this immune-modulatory approach into the clinical setting of PD.

## Results

### Assay simplification

**Figure 1:**
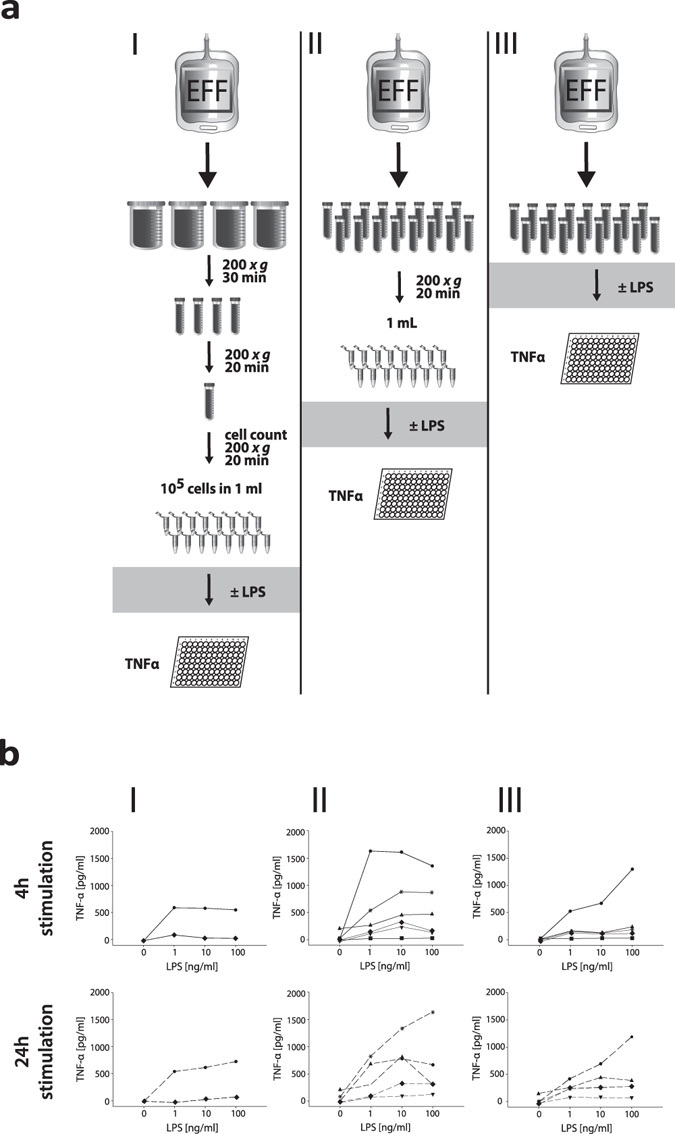
Bio-assay simplification. (**a**) Schematic of the simplification process for the *ex-vivo* stimulated cytokine release assay. Stimulations were performed as described in (I–III). (**b**) The left column (I) shows TNF-α release from a defined number of cells (10^5^ cells in 1 ml). The middle and right columns show TNF-α release from an undefined number of cells concentrated from a defined volume with (II) or without (III) prior centrifugation. In 3 patients insufficient cell number prevented stimulation of a defined number of cells, and in one patient this limitation permitted completion of only the 4 h stimulation condition.

Peritoneal cells for stimulation experiments were isolated by three increasingly simplified protocols (Fig. 1a) from PD effluents after 4 hour dwells from stable PD patients.

First, a defined cell number of PD effluent leukocytes (10^5^/ml) was stimulated with LPS (0–100 ng/ml) for 4 and 24 hours. Tumor necrosis factor alpha (TNF-α) release was measured in the cell-free supernatants. TNF-α was detectable in only 2 of 6 supernatants from stimulated cells, and in no supernatant from unstimulated control cells (Fig. 1b left column).

Second, a defined effluent volume (containing an *a-priori* unknown number of cells) was concentrated by centrifugation into 1 ml of medium. As shown in Fig. 1b (middle column) this procedure yielded measurable results in all patients. Secreted TNF-α in the cell-free supernatants showed a dose-dependent increase in response to exposure to increasing LPS concentrations over 4 hours, with further increases after 24 hours in some patients.

**Figure 2:**
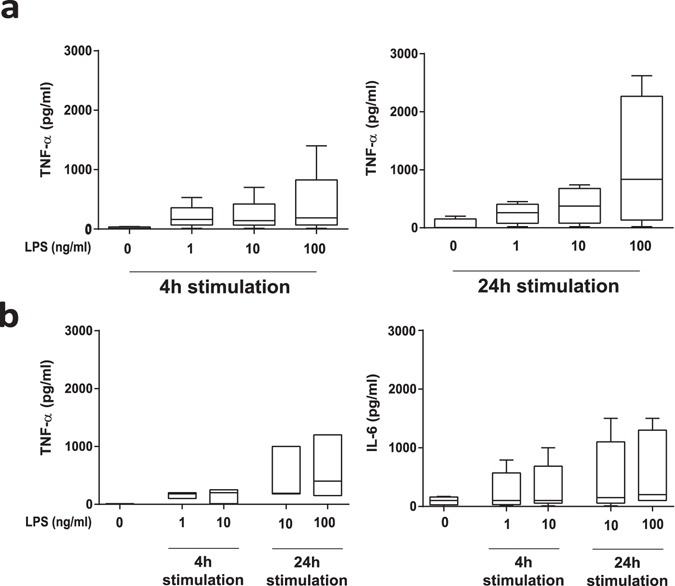
*Ex-vivo* stimulation assay. Levels of TNF-α and (b only) IL-6 in uncentrifuged PD effluent samples (4 h PET dwell) of 50 ml (**a**) initial cohort, n = 6) or 9 ml (**b**) independent validation cohort, n = 5) in volume, directly stimulated with LPS (0–100 ng/ml) at 37 °C for 4 or 24 h.

Third, to minimize artefacts from pre-analytic concentration steps, we directly stimulated cells present in a defined volume of fresh PD effluent. As shown in Fig. 1b in the right column, this approach resulted in LPS dose-dependent stimulation of TNF-α secretion comparable to those observed after the more complicated protocols – despite cell numbers 50 times lower than in samples concentrated by centrifugation. This third, simplest protocol was next repeated with 5 additional stable PD patients undergoing 4-hour routine peritoneal equilibration tests (PETs). Fresh effluents (50 ml and 9 ml) were stimulated with LPS (0, 1, 10 and 100 ng/ml for 4 and 24 hours), with reproducible, LPS concentration-dependent secretion of TNF-α and interleukin 6 (IL-6) (Fig. 2).

### Randomized cross-over feasibility trial

Based on the findings from the simplification steps, we next tested the feasibility of using the candidate protocol in samples of PD effluent obtained at different dwell times during PETs in an independent cohort of stable PD patients (Table 1). 9 ml samples of crude PD effluent were thus treated *ex-vivo* with the TLR4 agonist LPS (100 ng/ml), the TLR2 agonist Pam_3_Cys (100 ng/ml) or with both. IL-6 and TNF-α release were assessed in supernatants after 24 hours of stimulation as a functional surrogate test for global peritoneal immune-competence. The high correlation of cytokine values in samples drawn from the mixed effluent in immediate succession and treated in parallel also suggests that *ex-vivo* stimulated cytokine levels reflected peritoneal effluent leukocyte function, and that variations in stimulation and analysis were minimal.

**Table 1:** Baseline characteristics of the pilot RCT study population.

*Variable*	
Patients (n)	6
Sex (n) (male/female)	4/2
Age (y)	52.5 (45–68)
Body mass index (kg/m^3^)	23.90 (20.3–35.3)
Body surface area (m^2^)^a^	1.87 (1.73–2.01)
Time on PD (m)	17.8 (4.9–48.1)
Patients with residual renal output (n)	4
Residual renal urine output^b^ (ml)	2030 (1750–2500)
Residual renal clearance^b,c^ (ml/min/1.73 m^2^)	5.04 (3.2–8.3)
PD modality (n) (APD/CAPD)	5/1
Total weekly Kt/V (-)	2.39 (1.68–2.82)
History of previous peritonitis (n)	2
Transport type^d^ (n) (high/high average)	3/3

Data are presented as median (range). Body surface area was calculated with the Du Bois method. ^b^Median residual renal urine output and clearance were computed from 4 patients with residual renal output. ^c^Residual renal clearance was calculated as mean of renal creatinine and renal urea clearance using 24 h urine samples. ^d^Transport type was determined from the PET with standard 3.86% PDF.

**Figure 3:**
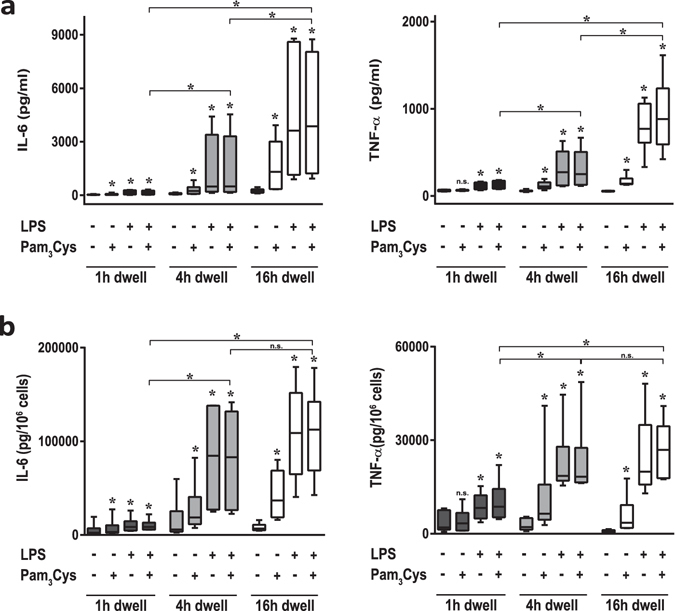
*Ex-vivo* stimulation assay in effluents of different PET dwell times. (**a**) Levels of IL-6 (left) and TNF-α (right) in 9 ml effluent samples of the indicated dwell times, after direct *ex-vivo* stimulation by LPS and/or Pam_3_Cys (100 ng/ml as indicated) for 24 h at 37 °C. Effluents from 6 patients of the randomized controlled trial without AlaGln supplementation. IL-6 and TNF-α release after maximum stimulation differed at all dwell times (*p < 0.05 vs. control per dwell time) (**b**) Measured cytokine levels as normalized per 10^6^ cells. IL-6 and TNF-α release were significantly higher at all dwell times (1, 4 and 16 h) after maximum stimulation with 100 ng/ml LPS plus 100 ng/ml Pam3Cys (*p < 0.05 vs. control per dwell time). After normalization for peritoneal cell count the difference between the 1 h dwell and the 4 h and 16 h dwells was significant (*p < 0.05), whereas cytokine release values at 4 h and 16 h were indistinguishable. Wilcoxon matched-pairs signed rank test was used to calculate *p*-values.

Stimulated cytokine release in effluent samples was significantly influenced by dwell time, with lower values for IL-6 and TNF-α release observed in the 1-hour dwell samples and higher values in the overnight dwell effluent samples (16 hours dwell time) (both *p* < 0.05 vs. 4-hour dwell) (Fig. 3a). Stimulated release of cytokines in effluent samples obtained at 4 hours of dwell time was increased to an extent comparable to that previously observed in the test optimization phase. Addition of Pam_3_Cys and LPS individually and in combination each significantly increased release of IL-6 and TNF-α (*p* < 0.05 *vs*. unstimulated). After normalization to effluent cell numbers, TLR-stimulated cytokine release from of effluent samples obtained at 1-hour dwell time remained significantly lower than stimulated values in effluent samples from later dwell times (Fig. 3a,b). This depressed TLR-stimulation of cell number-normalized cytokine release in samples obtained at the early dwell time (IL-6: 14.3% (IQR (interquartile range) 7.3–34.4); TNF-α: 32.9% (20.2–50.5) compared to cytokine release at the 4-hour dwell time) suggests the influence of dwell time-dependent factors, such as equilibration of PDF during the dwell.

This depressed cytokine release also led us to test the ability of the assay to discriminate immune-modulatory effects of AlaGln on *ex-vivo* stimulated cytokine release in PET-derived effluent samples obtained at early dwell time. Concentrations of TLR-ligands and exposure time (24 hours stimulation with a combination of LPS (100 ng/ml) and Pam_3_Cys (100 ng/ml)) were selected with the intent of providing maximal stimulation of cytokine expression. The 1-hour dwell of the PET was selected as the time of maximally depressed IL-6 release.

**Figure 4:**
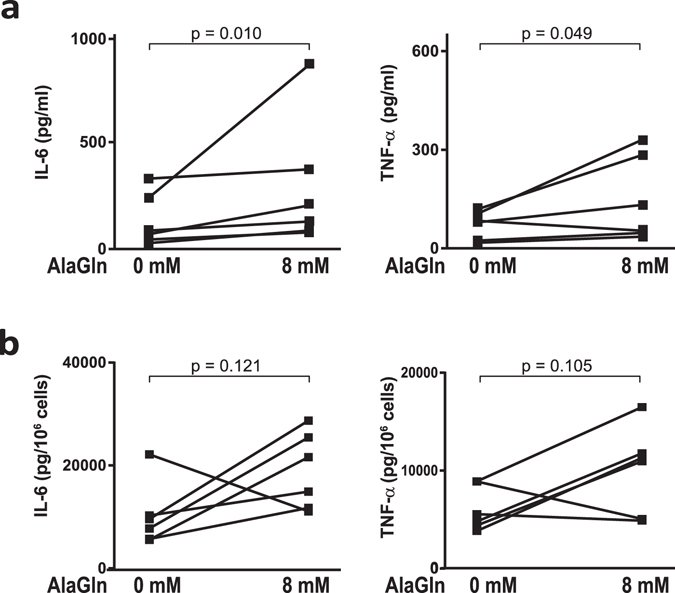
Effect of AlaGln supplementation on *ex-vivo* stimulated cytokine release. (**a**) Levels of IL-6 (left) and TNF-α (right) in 9 ml effluent samples of the 1 h PET dwell without (0 mM) or with (8 mM) AlaGln supplemented PDF. 9 ml effluent samples were directly stimulated with 100 ng/ml LPS plus 100 ng/ml Pam_3_Cys and incubated for 24 h at 37 °C. IL-6 release was significantly increased (p = 0.010) by addition of 8 mM AlaGln during the dwell (TNF-α, p = 0.0487). (**b**) Cytokine levels as normalized per 10^6^ cells. Data show results from all 6 patients of the randomized controlled trial with and without AlaGln supplementation. Paired t-test on log-transformed data was used to calculate *p*-values.

AlaGln addition to PDF prior to its instillation into the peritoneal cavity significantly increased median *ex-vivo* stimulated release of IL-6 from PD effluents more than two-fold (233.6% (IQR 113–359) *vs*. standard PDF, p = 0.010) in samples obtained at the 1-hour PET-dwell. AlaGln addition to PDF also improved TNF-α release at the 1-hour sampling time point of the PET almost two-fold (197.8% (65–298) *vs*. standard PDF, p = 0.0487). However, the effects of AlaGln supplementation on cell count-normalized stimulated cytokine release, though comparable in apparent magnitude (IL-6: 252% (IQR 50–389); TNF-α: 215% (57–295) of standard PDF), did not reach statistical significance (p = 0.121 and p = 0.105) due to disproportionate variation of cytokines and cell counts (Fig. 4).

AlaGln supplementation had no significant effect on routine clinical blood parameters, PD effluent laboratory parameters, peritoneal transport parameters (Table 2 and Suppl. Tab. 1), on PET effluent cell numbers and differentials, or on basal levels of IL-6, IL-8 and TNF-α (Table 2).

**Table 2:** PD effluent parameters.

	AlaGln 0 mM	AlaGln 8 mM	AlaGln 16 mM	*p*-value^a^	*p*-value^b^
**total cell count (G/L)**					
PET (4 h)	0.012 (0.002–0.032)	0.01 (0.004–0.03)	0.008 (0.004–0.061)	0.84	0.81
overnight dwell (16 h)	0.036 (0.015–0.06)	0.036 (0.022–0.1)	0.029 (0.015–0.339)	0.19	0.99
**ON-Differential (rel%)**					
monocytes/macrophages	90 (78–95)	90 (81–94)	87.5 (86–99)	0.81	0.81
lymphocytes	4.5 (2–19)	5.5 (2–11)	6 (0–12)	0.63	0.63
neutrophils	2.5 (0–10)	5.5 (1–10)	1.5 (0–7)	0.66	0.63
eosinophils	0 (0–2)	0 (0–1)	1.5 (0–5)	0.99	0.13
basophils	0 (0–1)	0 (0–1)	0 (0–1)	0.99	0.99
**Plasma IL-6 (pg/ml)** ^**c**^	6.5 (4.6–10)	7.9 (4.5–9.6)	6.4 (4.5–10.7)	0.84	0.99
**basal IL6 (pg/ml)**					
PET (1 h)	58 (37.4–82.4)	88.1 (38.9–124.8)	59 (50.4–128.1)	0.16	0.31
PET (4 h)	118.9 (79.5–138.8)	124.9 (61.7–148.4)	96.3 (70.0–151.4)	0.99	0.84
overnight dwell (16 h)	355.8 (176.2–426.4)	434.1 (197.5–632.9)	310.6 (250.4–1108)	0.31	0.56
**basal IL-8 (pg/ml)** ^**d**^					
PET (4 h)	9.7 (5.7–13.4)	7.7 (5.2–16.5)	7.8 (2.5–13.3)	0.84	0.22
overnight dwell (16 h)	12.5 (8.1–23.7)	16.3 (10.5–32.3)	16 (11.4–27.4)	0.16	0.16
**Clinical chemistry** ^**e**^					
Albumin loss (g)	1.3 (1.0–1.5)	1.3 (0.9–1.6)	1.3 (0.9–1.6)	0.99	0.88
Total protein loss (g)	1.8 (1.3–1.8)	1.6 (1.2–2.1)	1.4 (1.2–2.2)	0.63	0.86
Sodium dip (mmol/l)	5.5 (1–9)	4.0 (−1–7)	4.5 (1–6)	0.19	0.19
D_4 h_/D_0 h_ Glucose	0.20 (0.14–028)	0.22 (0.14–0.30)	0.27 (0.002–0.42)	0.50	0.63
D/P creatinine	0.85 (0.78–1.06)	0.82 (0.69–0.96)	0.82 (0.10–0.91)	0.63	0.16
D/P BUN (4 h)	1.03 (0.92–1.09)	1.00 (0.95–2.22)	0.99 (0.96–1.02)	0.99	0.22
D/P albumin (4 h)	0.01 (0–0.02)	0.02 (0.01–0.02)	0.01 (0.01–0.06)	0.53	0.94
D/P total protein (4 h)	0.01 (0–0.02)	0.01 (0.01–0.02)	0.01 (0–0.02)	0.63	0.69
CA 125 (kU/l)	37.0 (21.7–65.8)	39.5 (21.5–56.3)	37.4 (18.6–46.3)	0.81	0.16
Ultrafiltration (ml)	560 (30–1015)	484 (119–794)	469 (−141–931)	0.31	0.06

Data are represented as median (range) of all 6 patients enrolled in the feasibility trial. Wilcoxon matched-pairs signed rank test was used to calculate *p*-values. ^a^Comparison control without AlaGln versus 8 mM AlaGln in PDF. ^b^Comparison control without AlaGln versus 16 mM AlaGln in PDF. ^c^IL-6 was measured in EDTA plasma samples at the 2 h time point of the PET. ^d^Basal IL-8 was not detectable in 1 h PET sample. ^e^Dialysate parameters are measured at the 4 h PET time point. To evaluate the peritoneal permeability for small solutes, dialysate-to-plasma ratios (D/P) of creatinine, BUN, albumin and total protein were calculated from the 4 h effluent value and the plasma values at 2 hours. The permeability for glucose was estimated as the ratio between the dialysate glucose concentration at 4 h to that at 0 h dwell (D4/D0). As indirect measure of free water transport, the difference between dialysate sodium concentration at 1 h and that at baseline (sodium dip) was calculated. Uncorrected *p*-values are shown.

### Transcriptome analysis of peritoneal effluent cells from cross-over-treated PD patients

We next conducted transcriptome analysis of peritoneal effluent cells before *ex-vivo* LPS-stimulation for assessment of immune-competence. Our workflow combined RNA sequencing of mRNA and microarray analysis of miRNA.

After filtering out low-abundance transcripts, high quality reads could be assigned to 9797 unique genes. Non-supervised analysis of the data showed clustering of samples from individual patients, rather than clustering of all samples from individual treatment groups (Suppl. Fig. 1), suggesting that the inter-individual difference among patient transcriptomes is greater than the difference attributable to AlaGln supplementation of PDF. The clustering of patients from samples harvested from two separate PETs, separated by up to two months confirms reproducibility of the transcriptome analysis.

Supervised analysis of the paired data exploited the cross-over study design to identify effects of the treatment independent of inter-patient differences. Samples from the same patient treated without AlaGln served as control for the RNA samples derived from the PET dwell with AlaGln-supplemented PDF.

**Figure 5:**
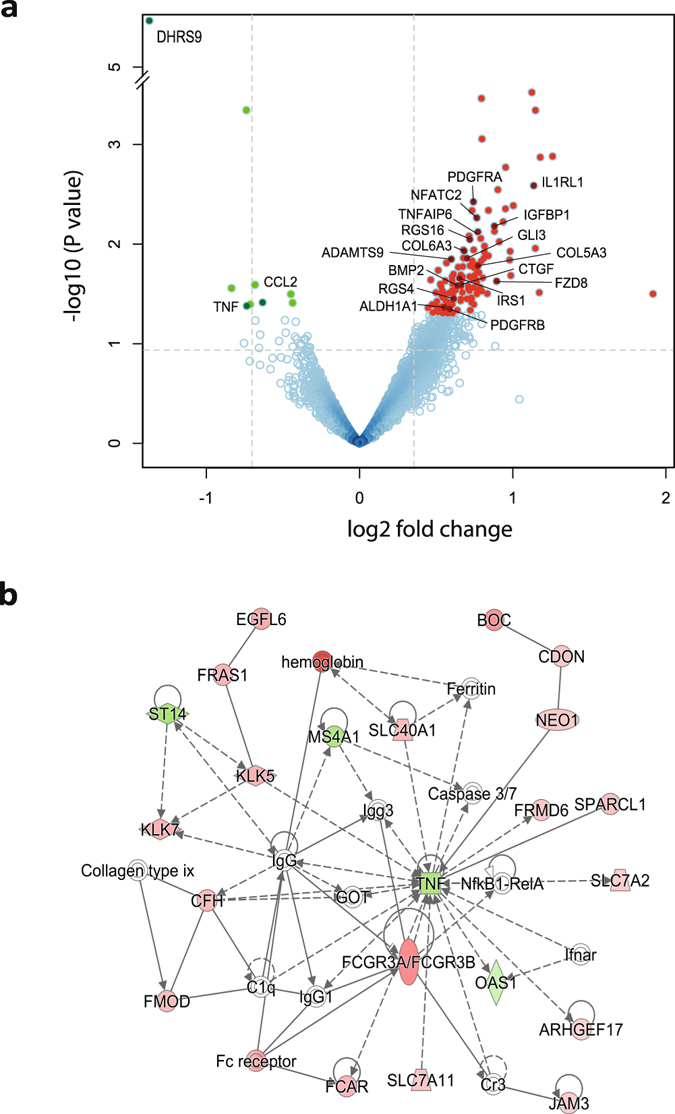
Network analysis of differentially abundant transcripts in peritoneal effluent cells. RNA-seq data was obtained from cells harvested from PET effluent samples in the randomized clinical trial testing the effect of supplemental AlaGln in PDF. (**a**) Volcano plot of abundance ratios and p-values of individual transcripts with and without AlaGln. Log fold-change values of the paired analysis with *vs*. without AlaGln are plotted against the negative log *p* value for individual transcripts (**b**). Interaction network generated from differentially expressed genes. Each node represents a gene and edges interaction among genes. The node color indicates up-regulation (red) or down-regulation (green) in the presence of added AlaGln.

Analysis based on a negative binomial model revealed differential abundance of 146 transcripts with *p*-value < 0.05 (Suppl. Tab. 2, Fig. 5a). AlaGln supplementation resulted predominantly in upregulation of transcripts, consistent with glutamine’s status as a conditionally essential amino acid, required for energy metabolism under stressful conditions. We conducted an IPA network analysis to cluster functionally related genes and identify the highest-ranked networks (encompassing the largest numbers of differentially expressed candidate genes from the transcriptomics analysis). Interestingly, the second largest network centers around the down-regulated TNF-α transcript (Fig. 5b). The other two top networks are related to Akt signaling and IL-1R signaling (Suppl. Fig. 2).

Because the fraction of false positive hits was unknown, the transcripts showing differential abundance were subjected to pathway analysis, providing a second level of evidence (Table 3). To evaluate the stability of the results, pathway analysis was also carried out with a set of 41 candidate genes which passed the stricter threshold of a *p*-value < 0.015 and fold-change > 1.5. Pathways that remain significantly enriched, even with this reduced set of genes, include IL-6 signaling and are indicated in bold in Table 3.

**Table 3:** Pathways enriched in the transcriptome of peritoneal effluent cells obtained from PET tests in the randomized trial with and without AlaGln.

Canonical Pathway	*p*-Value	Molecules^a^
**Hepatic Fibrosis**/**Hepatic Stellate Cell Activation**	4,68E-06	COL5A3↑, CTGF↑, COL6A3↑, CCL2↓, IL1RL1↑, PDGFRA↑, SERPINE1↑, TNF↓, PDGFRB↑
Noradrenaline and Adrenaline Degradation	1,58E-04	MAOB↑, ALDH1A1↑, DHRS9↓, ADH1B↑
Retinoate Biosynthesis I	1,58E-03	ALDH1A1↑, DHRS9↓, BMP2↑
Serotonin Degradation	1,66E-03	MAOB↑, ALDH1A1↑, DHRS9↓, ADH1B↑
Ethanol Degradation II	2,04E-03	ALDH1A1↑, DHRS9↓, ADH1B↑
Human Embryonic Stem Cell Pluripotency	3,02E-03	FZD8↑, IRS1↑, BMP2↑, PDGFRA↑, PDGFRB↑
**Axonal Guidance Signaling**	3,72E-03	FZD8↑, SEMA6D↑, GLI3↑, SEMA3D↑, ADAMTS1↑, IRS1↑, BMP2↑, NFATC2↑, ADAMTS9↑
**PPAR Signaling**	3,80E-03	IL1RL1↑, PDGFRA↑, TNF↓, PDGFRB↑
PAK Signaling	5,13E-03	IRS1↑, PDGFRA↑, TNF↓, PDGFRB↑
Role of Osteoblasts, Osteoclasts and Chondrocytes in Rheumatoid Arthritis	5,25E-03	FZD8↑, IL1RL1↑, IRS1↑, BMP2↑, NFATC2↑, TNF↓
Role of Macrophages, Fibroblasts and Endothelial Cells in Rheumatoid Arthritis	5,37E-03	FZD8↑, CCL2↓, IL1RL1↑, IRS1↑, NFATC2↑, TNF↓, FCGR3A/FCGR3B
Differential Regulation of Cytokine Production in Macrophages and T Helper Cells by IL-17A and IL-17F	6,61E-03	CCL2↓, TNF↓
MSP-RON Signaling Pathway	7,59E-03	CCL2↓, IRS1↑, TNF↓
NF-κB Signaling	7,94E-03	IRS1↑, BMP2↑, PDGFRA↑, TNF↓, PDGFRB↑
3-phosphoinositide Biosynthesis	9,12E-03	PTPN13↑, IRS1↑, PDGFRA↑, CILP↑, PDGFRB↑
Dendritic Cell Maturation	9,77E-03	COL5A3↑, DDR2↑, IRS1↑, TNF↓, FCGR3A/FCGR3B
PXR/RXR Activation	1,00E-02	ALDH1A1↑, IGFBP1↑, TNF↓
Differential Regulation of Cytokine Production in Intestinal Epithelial Cells by IL-17A and IL-17F	1,07E-02	CCL2↓, TNF↓
Putrescine Degradation III	1,07E-02	MAOB↑, ALDH1A1↑
Atherosclerosis Signaling	1,12E-02	ALOX15↑, COL5A3↑, CCL2↓, TNF↓
**IL-6 Signaling**	1,12E-02	TNFAIP6↑, IL1RL1↑, IRS1↑, TNF↓
Tryptophan Degradation X (Mammalian, via Tryptamine)	1,26E-02	MAOB↑, ALDH1A1↑
HMGB1 Signaling	1,32E-02	CCL2↓, IRS1↑, SERPINE1↑, TNF↓
Basal Cell Carcinoma Signaling	1,32E-02	FZD8↑, GLI3↑, BMP2↑
TREM1 Signaling	1,48E-02	CCL2↓, IL1RL1↑, TNF↓
**VDR/RXR Activation**	1,62E-02	WT1↑, IL1RL1↑, IGFBP1↑
Superpathway of Inositol Phosphate Compounds	1,95E-02	PTPN13↑, IRS1↑, PDGFRA↑, CILP↑, PDGFRB↑
Glioblastoma Multiforme Signaling	2,34E-02	FZD8↑, IRS1↑, PDGFRA↑, PDGFRB↑
PDGF Signaling	2,40E-02	IRS1↑, PDGFRA↑, PDGFRB↑
Dopamine Degradation	2,40E-02	MAOB↑, ALDH1A1↑
Gαq Signaling	2,40E-02	IRS1↑, RGS16↑, RGS4↑, NFATC2↑
Granulocyte Adhesion and Diapedesis	3,31E-02	CCL2↓, JAM3↑, IL1RL1↑, TNF↓
Role of Hypercytokinemia/hyperchemokinemia in the Pathogenesis of Influenza	3,47E-02	CCL2↓, TNF↓
IGF-1 Signaling	3,63E-02	CTGF↑, IRS1↑, IGFBP1↑
IL-9 Signaling	3,80E-02	IRS1↑, TNF↓
G-Protein Coupled Receptor Signaling	3,80E-02	PTGER3↑, IRS1↑, RGS16↑, RGS4↑, PDE4D↑
Glioma Signaling	3,98E-02	IRS1↑, PDGFRA↑, PDGFRB↑
p53 Signaling	4,07E-02	WT1↑, THBS1↑, IRS1↑
Agranulocyte Adhesion and Diapedesis	4,07E-02	CCL2↓, JAM3↑, CD34↑, TNF↓
Regulation of the Epithelial-Mesenchymal Transition Pathway	4,07E-02	FZD8↑, IRS1↑, JAG1↑, PDGFRB↑
Glucocorticoid Receptor Signaling	4,68E-02	CCL2↓, IRS1↑, NFATC2↑, SERPINE1↑, TNF↓
Renin-Angiotensin Signaling	4,90E-02	CCL2↓, IRS1↑, TNF↓
Docosahexaenoic Acid (DHA) Signaling	4,90E-02	ALOX15↑, IRS1↑
LXR/RXR Activation	5,01E-02	CCL2↓, IL1RL1↑, TNF↓

Analysis of PD effluent cells of the 4 h PET time point. ^a^Comparison control without AlaGln versus 8 mM AlaGln in PDF. ↑ up regulated molecules, ↓ down regulated molecules

Microarray-based analysis of small non-coding RNAs was carried out to increase transcriptome coverage and gain additional functional insight. After filtering out low quality signals, 1548 miRNAs were quantified. Of these, 109 were identified as differentially abundant (*p* < 0.05), and 13 remained significant using the stricter threshold of *p* < 0.01 and fold-change > 1.5. (Suppl. Fig. 3 and Suppl. Tab. 3)

**Figure 6:**
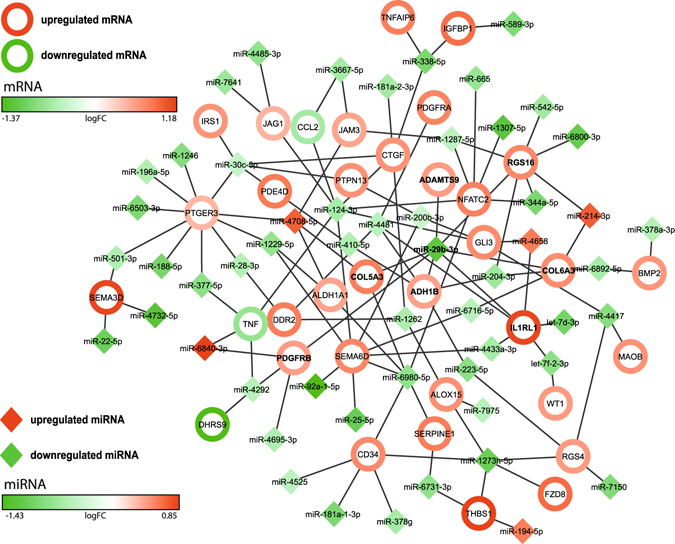
Analysis of miRNA-mRNA interaction in peritoneal effluent cells. Interaction network of differentially abundant miRNAs (solid diamonds) and differentially abundant mRNAs (unfilled circles) present in significantly enriched pathways. The top candidate miR-29b-3p (bold in center) is down-regulated and links to 7 up-regulated genes (bold). Table 4 presents a rank list of interactions, based on number of observed and concordantly regulated target genes.

Interactions between mRNAs and miRNAs were detected by TargetScan and the IPA software. Eighty-four of the 109 non-coding RNAs with differential abundance were mapped to miRNAs in IPA (25 were nominal non-coding RNAs yet uncharacterized in the IPA repository; see Suppl. Tab. 3 for mapping status). The search for miRNA:mRNA interaction yielded 418 hits of which 117 could be assigned to genes included in significantly enriched pathways (see Table 3 for genes; Suppl. Tab. 4 presents all miRNA:mRNA interactions). The miRNA:mRNA interactions associated with enriched pathway genes are visualized as a network (Fig. 6b). Increased levels of specific miRNAs should reduce both expression and upregulation of the target genes of those miRNAs, and vice versa. Interestingly, the majority of identified miRNAs are down-regulated by AlaGln, consistent with up-regulation of the target genes of those miRNAs, as indicated by opposing color-gradients in the network. Table 4 lists AlaGln-regulated miRNAs with known regulatory relationship to more than one gene within significantly enriched pathways. Table 4 also lists the target genes of those miRNAs and how those target genes are regulated. The down-regulated miRNAs miR-29b, miR-124, miR-6980, miR-1273h and miR-30c each are associated with at least 5 concordantly up-regulated target genes, with the only observed exceptions being the down-regulation of CCL2 and TNF. Thus, these five miRNAs can be regarded as signaling hubs of AlaGln treatment-associated transcriptional regulation.

**Table 4:** Micro-RNAs (miRNAs) influencing more than one gene within pathways enriched in cells from AlaGln-supplemented PDF

miRNA	miRNA FC	miRNA direction	Genes in pathways (N)	Regulated Genes	Target gene regulation
miR-29b	−2.32	down	7	ADAMTS9↑, ADH1B↑, COL5A3↑, COL6A3↑, IL1RL1↑, PDGFRB↑, RGS16↑	7 up, 0 down of 7
miR-124	−1.56	down	7	CCL2↓, CTGF↑, GLI3↑, JAG1↑, NFATC2↑, SEMA6D↑, TNF↓	5 up, 2 down of 7
miR-6980	−1.73	down	6	ALDH1A1↑, CD34↑, COL5A3↑, NFATC2↑, SEMA6D↑, SERPINE1↑	6 up, 0 down of 6
miR-1273h	−2.15	down	5	ALOX15↑, CD34↑, FZD8↑, RGS4↑, THBS1↑	5 up, 0 down of 5
miR-30c	−1.33	down	5	CTGF↑, IRS1↑, PDE4D↑, PTGER3↑, PTPN13↑	5 up, 0 down of 5
miR-4481	−1.41	down	4	ADH1B↑, DDR2↑, IL1RL1↑, PDGFRA↑	4 up, 0 down of 4
miR-4708	1.61	up	4	ALDH1A1↑, COL5A3↑, JAM3↑, PTGER3↑	4 up, 0 down of 4
miR-1229	−1.71	down	3	ALDH1A1↑, PTGER3↑, SEMA6D↑	3 up, 0 down of 3
miR-1262	−1.52	down	3	ALOX15↑, CTGF↑, DDR2↑	3 up, 0 down of 3
miR-1287	−1.40	down	3	JAM3↑, NFATC2↑, RGS16↑	3 up, 0 down of 3
miR-200b	−1.30	down	3	GLI3↑, PTPN13↑, SEMA6D↑	3 up, 0 down of 3
miR-204	−1.63	down	3	ADH1B↑, COL6A3↑, RGS16↑	3 up, 0 down of 3
miR-338	−1.97	down	3	CTGF↑, IGFBP1↑, TNFAIP6↑	3 up, 0 down of 3
miR-4417	−1.85	down	3	GLI3↑, MAOB↑, RGS4↑	3 up, 0 down of 3
miR-4292	−1.43	down	3	DHRS9↓, PDGFRB↑, TNF↓	1 up, 2 down of 3
let-7f-2	−1.77	down	2	IL1RL1↑, WT1↑	2 up, 0 down of 2
miR-223	−1.80	down	2	ADH1B↑, RGS4↑	2 up, 0 down of 2
miR-28	−1.46	down	2	DDR2↑, PTGER3↑	2 up, 0 down of 2
miR-344a	−1.97	down	2	NFATC2↑, RGS16↑	2 up, 0 down of 2
miR-410	−1.50	down	2	ADH1B↑, PDE4D↑	2 up, 0 down of 2
miR-4433a	−1.56	down	2	IL1RL1↑, SEMA6D↑	2 up, 0 down of 2
miR-501	−1.49	down	2	PTGER3↑, SEMA3D↑	2 up, 0 down of 2
miR-6716	−1.42	down	2	COL6A3↑, SEMA6D↑	2 up, 0 down of 2
miR-6731	−1.75	down	2	SERPINE1↑, THBS1↑	2 up, 0 down of 2
miR-6892	−1.55	down	2	BMP2↑, COL6A3↑	2 up, 0 down of 2
miR-3667	−1.57	down	2	CCL2↓, JAM3↑	1 up, 1 down of 2
miR-377	−1.77	down	2	PTGER3↑, TNF↓	1 up, 1 down of 2
miR-6840	1.80	up	2	PDGFRB↑, TNF↓	1 up, 1 down of 2
miR-214	1.62	up	2	COL6A3↑, RGS16↑	2 up, 0 down of 2
miR-4658	1.49	up	2	GLI3↑, IL1RL1↑	2 up, 0 down of 2

Analysis of PD effluent cells of the 4 h PET time point. Comparison control without AlaGln versus 8 mM AlaGln in PDF. ↑ up/↓ down regulated genes.

## Discussion

In sepsis diagnosis and research, *ex-vivo* stimulated cytokine release assays have long been recognized as useful tools to determine immune-competence^15–18^. LPS-stimulated cytokine release of whole blood has been successfully applied as a test for predicting clinical outcomes in intensive care unit patients post-surgery and/or trauma^16, 24, 25^. More than 10 years ago *ex-vivo* stimulated cytokine release was introduced to measure the function of peritoneal leukocytes. This test examined mainly macrophages isolated either from chilled overnight PD effluent or from fresh effluent from short dwells, and assessed cytokine secretion after TLR-stimulation by LPS or by serum-treated zymosan (STZ)^6, 11, 22, 23, 26^. However, this test has not yet achieved broad acceptance in PD outcome research, likely due to its impracticality in the clinical setting, to uncertain transferability from other disease models, and to incomplete understanding of underlying molecular mechanisms.

The first part of our study thus set out to simplify this test by assessing *ex-vivo* stimulated cytokine release directly in crude PD effluents. Original procedures, i.e. stimulation of a defined number of cells following pre-analytic isolation, counting and concentration steps, were feasible only in a subgroup of stable patients. In contrast, stimulation of cells in a defined volume of effluent, with or without prior concentration by centrifugation, allowed assessment of *ex-vivo* stimulated cytokine release in all patients. Interestingly, absolute level of cytokine release remained almost unchanged despite the (~50-fold) lower cell density due to omission of pre-analytic concentration steps. This finding suggests that procedures for effluent cell isolation and/or concentration themselves dampened cell reactivity, and should thus be eliminated.

As with whole blood stimulation, the volume of PD effluent rather than the number of responding cells was therefore held constant in the assay further developed in this study. Although adding inter-individual variation among, and intra-individual within PD patients over time, this may nonetheless amplify the diagnostic value of the measurements. Per-cell normalization for peritoneal effluent cell number in each sample, however, may be a challenge, as the type of responding cells cannot be adequately determined without reducing clinical feasibility^27^. Variation of the cell count – if disproportionate in magnitude to the cytokine release – can lead to an apparently inconsistent trend as assessed by (non-normalized) cytokine levels. We therefore decided for the remainder of the study to assess and report both global and per-cell normalized immune responses. Direct measurement of global (rather than normalized) cytokine release in fresh peritoneal effluent exposed to TLR ligands (mimicking bacterial challenge) may be regarded as a composite surrogate outcome reflecting immune-competence in the peritoneal cavity. Certainly, cohort studies in larger PD collectives are needed to compare the utility of each readout of this test for prediction of risk of infectious complications/peritonitis in PD^28^.

The next step in this study was to confirm the technical feasibility of the streamlined assay in a small independent PD population, and to obtain first evidence of its ability to discriminate sample dwell time and PD exposure to AlaGln-supplemented PD fluids.

The assay revealed that global responses of PD effluent samples to TLR-stimulation were dwell time-dependent. The shortest dwell time resulted in lowest cytokine release from stimulated samples. As previously described for whole blood culture-based tests, the observed variation between samples obtained at different dwell times was in part due to variability in leukocyte counts, with numbers of responding cells increasing as dwell time increased. This variation, however, will likely have no relevance in the clinical application of this test, as sampling will ultimately be restricted to a single, well defined PET time point. For any given dwell time, the variability of peritoneal cell number was within the range previously shown in whole blood cultures to remain informative^15^.

When normalized for cell count, the effluent sample collected at the shortest dwell time still demonstrated the lowest increases in IL-6 and TNF-α upon TLR-stimulation. Dampened cellular responsiveness towards TLR-stimulation during earlier dwell times suggest the importance of additional factors possibly intrinsic to the PD effluent, as the non-physiologic composition of PDF was less well equilibrated at early dwell times^6^. This early time window after dwell initiation might also be most relevant for an effective peritoneal host defence to stop the transition from bacterial contamination (*e.g*. from dialysate contamination by handling error) to peritonitis.

The known protective effects of glutamine against infectious complications in immune compromised hosts^20, 29^ prompted our assessment of PDF supplementation with AlaGln. Our simplified assay found significant effects of AlaGln supplementation on global IL-6 release in PD samples obtained from the early sampling time point of the PET, reflecting improved function of immune-competent peritoneal cells. Glutamine is an essential substrate for rapidly dividing cells of the immune system, and has been reported to increase numbers and improve function of lymphocytes and macrophages in varied *in-vitro* and *in-vivo* settings^30, 31^. Glutamine deficiency (glutamine levels markedly below normal serum levels) as present in the peritoneal cavity particularly during early dwell times with standard PDF, has been previously associated with increased vulnerability to stress through inadequate cellular stress response, impaired metabolism and immune-competence^32^. Our results in this PD pilot trial are concordant with data from a recent first-in-man trial that described significantly enhanced LPS-stimulated cytokine release from peripheral blood mononuclear cells following incubation in PD effluent from patients treated with AlaGln^33^. Interestingly, the relative effects of AlaGln-supplementation on cytokine responses in these trials appeared comparable to those previously reported (between 70 and 260%) comparing double-chamber bag PDF to a standard single-chamber bag PDF^6, 11, 22, 23, 26^. Infection rates and patient outcomes among the critically ill are known to deteriorate in parallel with glutamine depletion and improve upon glutamine supplementation^20, 34–36^. The degree of reduction in LPS-stimulated TNF-α production in cells from septic trauma patients was correlated with non-survival, whereas supplementation of parenteral nutrition with glutamine-containing dipeptides restored LPS-stimulated cytokine release following post-operative immune suppression^16, 19^. Additional studies with a larger population of PD patients will be required to validate the clinical impact of peritoneal immune-modulation by addition of AlaGln to PDF.

To search for molecular mechanisms underlying the enhanced response to TLR-stimulation, the transcriptomes of peritoneal effluent cells harvested from PD patients undergoing randomized exposure to PDF or AlaGln-supplemented PDF were analyzed by next-generation sequencing-based RNA-seq for mRNA and by microarray-based analysis of miRNA. Until now, there are hardly any studies presenting transcriptome analysis of patient derived PD effluent cells. This is the first study that uses highly sensitive RNA-seq which allows system-wide characterization of biological processes and pathways in miniscule amounts of cells, harvested from clinical PD effluents. We identified transcripts from nearly 10,000 unique genes, and data obtained from PET samples separated by at least one month showed good reproducibility. The fact that an individual patient can be identified in the data by a characteristic transcriptomic signature also suggests that delineation of biomarker signatures from omics data might be a promising option for a precision medicine approach in PD.

Pathway analysis of differentially abundant transcripts identified pathways attributable to stress responses, inflammation and immune-competence, fibrosis, and other pathways including peritoneal effluent cell biological processes not yet described in literature. In this study, we focused on pathways linked to immune signaling that might be highly relevant for peritoneal effluent cells. In particular, pathways linked to IL-6 signaling, to the IL-1 receptor and to IL-17 signaling were enriched in our dataset. Transcriptome analysis was repeated with more stringent cut-offs, in order to challenge the robustness of this analysis. Among those pathways that remained significantly enriched independent of number of input molecules was the IL-6 pathway, of specific interest since IL-6 is one of the reporter molecules of immune-competence in the *ex-vivo* TLR-stimulation assay. These results suggest that the responsiveness of peritoneal effluent cells upon maximal stimulation are primed by AlaGln addition. Therefore, our transcriptome data relate the functional results obtained with the streamlined assay to molecular mechanisms underlying enhanced response to TLR-stimulation. Unlike elevated basal levels of peritoneal IL-6 in PD patients, which predispose to membrane transformation^37, 38^, increased response to bacterial stimuli would more likely reduce peritonitis rates. However, further large scale trials are required to elucidate whether findings obtained in the settings of sepsis and other disorders can be translated to PD.

Several of the differentially regulated genes within the identified inflammation and immune-response pathways corroborate with modulation of responsiveness to immune stimuli by AlaGln. Although similar effects have been described in the context of glutamine supplementation in other models, the precise mechanism is still unclear^39^. As suggested by our data, the connection with G-protein signaling through regulator of G-protein signaling 4 (RGS4) and RGS16 might modulate leukocyte responsiveness^40–42^. G-protein signaling and its connection to the T_H_17 response might also link immune-modulatory effects of AlaGln and its recently reported anti-fibrotic properties^43^. Notably, although AlaGln treatment up-regulated the majority of differentially abundant transcripts, those down-stream of IL-17 (CCL2 and TNF-α) were down-regulated by AlaGln, with TNF-α being at the center of one of the top identified interaction networks, consistent with mitigation of acute inflammation, a known immune-modulatory effect of glutamine^39^. IL-17 activation was recently also linked to the pro-fibrotic inflammatory state associated with *in-vivo* PDF exposure. This effect was abrogated by AlaGln addition to PDF^43^. Confirmation of long-term effects on IL-6/IL-17 signaling-related increased immune-competence will require treatment of larger numbers of patients over more extended periods.

We also analyzed non-coding RNAs with a focus on miRNAs. miRNAs are regulators of transcription and/or translation of multiple target gene products that are in turn subject to regulation by multiple miRNAs. The resulting complexity of interaction networks allows identification of important hubs of transcriptional regulation. The majority of miRNAs identified as differentially abundant were down-regulated. Concordantly, differentially expressed potential mRNA targets of these miRNAs were upregulated, consistent with functional interaction of the miRNAs and their target genes. The node with the highest number of targets regulated as predicted within enriched pathways was miR-29b. miR-29b related enriched pathways included genes related to IL-1R signaling, G-protein-signaling, Akt signaling and extracellular matrix processes. IL-1R signaling has been shown to mediate sterile inflammation in mesothelial cells^44^. Circulating miR-29b and miR-21 have been proposed as biomarkers of PD homeostasis, as well as to join IL-6 in signaling between cancer cells and immune cells in co-culture^45, 46^. Our multi-level transcriptomics data are therefore consistent with peritoneal immune-modulation, at least for short-term administration of AlaGln during two dwells.

In conclusion, our study has shown that measurement of *ex-vivo* stimulated cytokine release can be performed by a simple assay in fresh peritoneal effluent samples in the clinical setting of PD. This method for assessment of function of peritoneal immune-competent cells was found to be reproducible and stable in repeated short studies of cross-over design, suggesting that relatively small sample sizes may suffice to allow detection of statistically meaningful effects of novel interventions in clinical trials. Addition of AlaGln to PDF primed immune-modulatory transcriptional pathways and increased immune function responsiveness, while dampening effector transcripts. However, future studies in larger populations will be needed to investigate the clinical usefulness of this assay to predict the risk for infectious complications in PD patients, and to further assess and understand peritoneal and systemic immune-modulatory effects of prolonged use of AlaGln-supplemented PDF.

## Material and Methods

Standard chemicals were purchased from Sigma-Aldrich (St.Louis, MO, USA) if not specified otherwise. Volumes of collected PD effluent were in excess of diagnostic requirements. The study was approved by the ethics committee of the Medical University of Vienna (EK1755/2012 and EK 2035/2015).

### Simplification of the *ex-vivo* stimulation protocol

PD effluents from 11 PD patients (5 male and 6 female; mean age 55.7 years, (26.8–79.6)) free of peritonitis for > 2 months and followed at the Department of Nephrology, Medical University of Vienna, Austria, were collected during a routine peritoneal equilibration test (PET) with commercial glucose-based PDF (Physioneal®; 3.86% glucose concentration, Baxter (Deerfield, IL, USA)). Aliquots of PD effluents were either used directly or after centrifugation at 200 × *g* for 20 or 30 minutes (Fig. 1a). Cell counts were calculated using a Cellometer® Auto T4 (Nexcelom Bioscience LLC, MA, USA). For *ex-vivo* stimulation with LPS (0–100 ng/ml) the PD effluent samples were prepared according to the following approaches: (I) concentration of peritoneal cells of 1 L PD effluent via centrifugation and stimulation of a defined peritoneal cell count of 10.000, in 1 ml culture medium (RPMI 1640, E15-048, PAA Laboratories GmbH, Pasching, Austria) (II) concentration of peritoneal cells via centrifugation of 50 ml PD effluent in 1 ml culture medium (RPMI 1640) (III) stimulation of 50 ml or 9 ml crude PD effluent. Following stimulation for 4 h and 24 h at 37 °C samples were centrifuged 200 × *g* for 10 minutes and supernatants were stored at −80 °C until further analyses. TNF-α was measured using a commercially available enzyme-linked immunosorbent assays (Human TNF alpha ELISA Ready-SET-Go, eBiosciences, Vienna, Austria) according to the manufacturer’s protocol.

### Randomized cross-over feasibility trial

The randomized cross-over pilot study (EudraCT 2012-004004-36, registered December 3, 2012) was conducted at the Medical University of Vienna to test the feasibility of the candidate protocol. The study was approved by the ethics committee of the Medical University of Vienna and was carried out in accord with the Declaration of Helsinki. All patients gave written informed consent. Positive feasibility was prospectively defined as at least five out of six patients with a full analysis set of *ex-vivo* stimulated cytokine release data across all treatment conditions. Patients on PD aged ≥ 19 years were included if they were stable for at least 2 months (absence of peritonitis or severe concomitant disease). Exclusion criteria included known hypersensitivity to or treatment with another investigational drug, presumed non-compliance, limited efficacy of PD, clinically significant inflammation, body weight < 50 kg, or immune-suppressive therapy. One patient among 7 screened subjects was not enrolled due to kidney transplantation before initiation of the trial.

Six clinically stable PD patients (4 male, 2 female) aged 52.5 (45–68) years, treated with PD for 17.8 (4.9–48.1) months, with total Kt/V of 2.39 (1.68–2.82) were included. Two patients had a history of peritonitis > 2 months before enrolment. 4 patients had residual renal function (Table 1). Patients were randomly allocated to cross-over treatment sequences by envelope randomization. Treatments were separated by a wash-out period ( ≥ 28 days).

For AlaGln treatment, 17.4 ml or 34.8 ml Dipeptiven® (200 mg AlaGln/ml, Fresenius-Kabi, Bad Homburg, Germany) was added to 2.0 L of PDF (Physioneal® 40 with 3.86% glucose, Baxter) immediately before instillation, resulting in a final AlaGln concentration of 8 or 16 mM AlaGln ( = 0.17% or 0.34%). The increase in total osmolality of the modified PDF was less than 1%. The control treatment was carried out with unmodified PDF (Physioneal® 40 with 3.86% glucose, Baxter). Two PD exchanges were performed with the respective test fluids, with an overnight dwell starting at 5 pm the day before a 4-hour peritoneal equilibration test (PET) starting at 9 am (total treatment time 20 h). Biochemical measurements and transfer kinetics between peritoneal and systemic circulation were analyzed in routine serum (at 2 hours of PET) and dialysate specimens. Values were compared using Wilcoxon matched-pairs signed rank test or paired t-tests of log-transformed cytokine and transcript data. Due to the exploratory character of the study, no adjustment for testing multiple outcomes and multiple interactions was performed. Reported *p*-values are results of two-sided tests.

Due to the results of the REDOXS trial^47^ published after the start of our trial, we decided to analyze routine clinical and laboratory data (“safety”) in the whole population, but did not perform analysis of *ex-vivo* stimulated cytokine release in the high dose treatment arm due to low likelihood for translation into clinical application.

For *ex-vivo* stimulation, peritoneal effluent was collected in 9 ml collection tubes with no additives (Vacuette®, greiner bio-one, Kremsmünster, Austria) from the overnight dwell, after 1 hour and 4 hours of the PET. The *ex-vivo* stimulation was carried out directly in the collection tubes as described below.

### Adverse event monitoring in the randomized pilot trial

None of the 8 adverse events in 4 individuals was classified as related to the administration of AlaGln. Three events occurred with AlaGln (1 with 8 mM: increase of blood pressure (2d post 8 mM PET), 2 with 16 mM: vertigo, periodontitis (14d post 16 mM PET)) and five without AlaGln. None of the adverse events was classified as severe.

### Cell isolation

Peritoneal cells were isolated from overnight and PET-effluents by centrifugation 200 × *g* for 30 minutes, cells were counted and cytospin slides (20.000 cells per slide) were prepared. Cytospins were fixed and stained with eosin and azure (Hemacolor, Merck Millipore, Darmstadt, Germany). Differential cell counts were performed in a blinded manner.

### Cytokine levels and *ex-vivo* stimulated cytokine release

Measurement of basal peritoneal levels of cytokines (interleukin 6 (IL-6) and interleukin 8 (IL-8) in effluent was carried out using the Immulite® system (Siemens Healthcare, Vienna, Austria) according to manufacturer’s instructions.

For stimulation, the toll-like receptor 4 (TLR4) agonist LPS (Lipopolysaccharides from *Escherichia coli* 055:B5, L6529) and/or the TLR2 agonist Pam_3_Cys (synthetic peptide S-[2,3-Bis(palmitoyloxy)-(2-RS)-propyl]-N-palmitoyl-(R)-Cys-(S)-Ser-Lys4-OH x 3 HCl, EMC microcollections, Germany) were used in concentrations of 1, 10 and 100 ng/ml for LPS and 100 ng/ml for Pam_3_Cys. Control cells were left unstimulated. Following incubation at 37 °C for 4 and/or 24 hours, samples were centrifuged at 200 × *g* for 10 minutes and supernatants were stored at −80°C until further analyses. Release of TNF-α and IL-6 was quantified in appropriate dilutions of stimulated and control supernatants by commercially available anti-human TNF-α and IL-6 ELISAs (eBioscience) per manufacturer’s instructions.

Values were compared using Wilcoxon matched-pairs signed rank test. Reported *p*-values are results of two-sided tests and were considered to be statistically significant if *p* < 0.05. Statistical analyses were performed using Prism 6 (GraphPad, La Jolla, CA, USA).

### Illumina RNA sequencing (RNA-seq) analysis

Fresh peritoneal effluent cells were harvested at 4 hours of the PETs from the patients in the randomized cross-over feasibility trial described above. RNA was isolated from cell pellets using Qiazol buffer and the miRNeasy Mini Kit (Qiagen) per manufacturer’s protocol. One patient yielded insufficient RNA under one condition and was therefore excluded from paired analysis. RNA was checked for integrity with the Agilent Bioanalyzer 2100 and subjected to transcriptome analysis by next generation RNA-seq (Illumina TruSeq mRNA with single-end 50 bp read length on a HiSeq 2000 (Illumina, Inc., San Diego, CA, USA)) per manufacturer’s protocol.

The Raw sequencing data was processed to remove any adaptor, PCR primers and low quality transcripts using FASTQC and Trimomatic softwares, providing comprehensive estimates of sample quality on the basis of read quality, read length, GC content, uncalled bases, ratio of bases called, sequence duplication, adaptor and PCR primer contamination. Resulting high quality, clean reads were aligned against the hg19 reference human genome assembly using tophat2 and bowtie2 packages (http://tophat.cbcb.umd.edu/). Gene expression measurement was performed from aligned reads by counting unique reads. The read count based gene expression data was normalized on the basis of library complexity and gene variation. The normalized and log transformed count data was compared among groups in a paired manner using a negative binomial model to identify differentially expressed genes. The differentially expressed genes were identified on the basis of raw *p-*value and fold-change. Genes were considered significantly differentially expressed if the *p*-value was <0.05 and absolute fold change >1.5. Raw RNA-seq data were submitted to ArrayExpress under the accession E-MTAB-5462.

Ingenuity Pathway Analysis (IPA 7.0, Qiagen, http//www.ingenuity.com) was used to identify pathways and interaction networks significantly affected by differentially expressed genes, calculating a *p-*value for each functional pathway using a one-tailed Fisher exact test. Pathways with p-values < 0.05 were considered significantly affected. For each network, IPA calculates a score derived from the *p*-value of one-tailed Fisher exact test [score = −log(p-value)] and indicates the likelihood of focus genes appearing together in the network due to random chance.

### miRNA data-analysis

Analysis of miRNAs was performed using the Affymetrix miRNA 4.0 array. Preparation of cDNA, hybridization to the array and scanning were per manufacturer’s protocols. Scanned array image quality was determined by standard Affymetrix metrics including background values, boxplots, MA plots and signals of spike in controls and were normalized with the Robust Multichip Average (RMA) algorithm (that includes background correction, quantile normalization and summarization transcript signal using the median polish algorithm). To identify differentially expressed genes, a linear model was implemented using linear model microarray analysis software Bioconductor R-package (LIMMA)^48^. In LIMMA, all probes were ranked by t statistic using a pooled variance, a technique particularly suited to small numbers of samples per group. The differentially expressed probes were identified on the basis of absolute fold change and raw P value calculated in paired as well as unpaired models. Raw miRNA array data were submitted to ArrayExpress under the accession E-MTAB-5461.

## Electronic supplementary material

Supplementary Material

Supplementary Table 2

Supplementary Table 3

Supplementary Table 4
