# Supplementary material for: Functional and Transcriptomic Characterization of Peritoneal Immune-Modulation by Addition of Alanyl-Glutamine to Dialysis Fluid
Source: Sci Rep. 2017 Jul 24;7:6229. doi: 10.1038/s41598-017-05872-2 (PMC5524796; doi:10.1038/s41598-017-05872-2)
Supplement: Supplementary file 1 — Supplementary Material [file 41598_2017_5872_MOESM1_ESM.pdf]

Supplementary Material

Functional and Transcriptomic Characterization of Peritoneal Immune-Modulation by Addition of Alanyl-Glutamine to Dialysis Fluid.

Rebecca Herzog[1, 2], Lilian Kuster[1], Julia Becker[1], Tobias Gluexam[1], Dietmar Pils[3], Andreas Spittler[4], Manoj K. Bhasin[5,6], Seth L. Alper[6,7], Andreas Vychytil[8], Christoph Aufricht[1], Klaus Kratochwill[1, 2] *

[1] Medical University of Vienna, Department of Pediatrics and Adolescent Medicine, Vienna, Austria

[2] Medical University of Vienna, Christian Doppler Laboratory for Molecular Research in Peritoneal Dialysis, Vienna, Austria

[3] Medical University of Vienna, Center for Medical Statistics, Informatics, and Intelligent Systems, Vienna, Austria

[4] Medical University of Vienna, Department of Surgery, Research Laboratories & Core Facility Flow Cytometry, Vienna, Austria

[5] Division of Interdisciplinary Medicine, Beth Israel Deaconess Medical Center, Boston, MA, USA

[6] Department of Medicine, Harvard Medical School, Boston, MA, USA

[7] Division of Nephrology and Center for Vascular Biology Research, Beth Israel Deaconess Medical Center, Boston, MA, USA

[8] Medical University of Vienna, Department of Medicine III, Division of Nephrology and Dialysis, Vienna, Austria

*__Corresponding author:__

Priv.Doz. DI Dr. Klaus Kratochwill
Division of Pediatric Nephrology and Gastroenterology, Department of Pediatrics and Adolescent Medicine, Medical University of Vienna
Währinger Gürtel 18-20, AT-1090 Vienna
Phone: +43/1/40400-73747
Fax: +43/1/40400-73598
E-mail: klaus.kratochwill@meduniwien.ac.at

Supplementary Figure 1

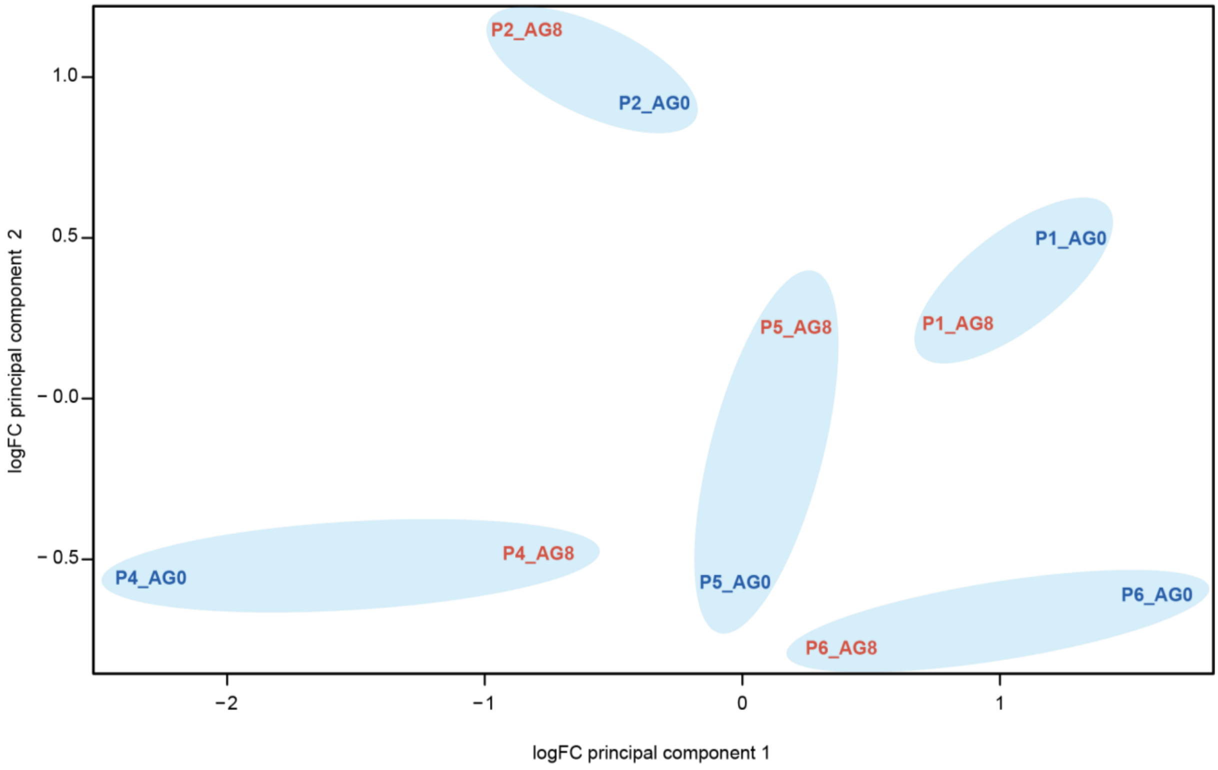

Principal component analysis of RNA-seq data obtained from effluent cells from PETs in the randomized clinical trial. The plot depicts the overall relationship of samples on the basis of similarity in transcriptome profile of samples. The X and Y-axes represent primary and secondary variables of highest and second-highest variance in data, respectively. Samples from any individual patient (P1-P6) treated with (AG8) and without (AG0) AlaGln are clustered, indicating good reproducibility between samples obtained at different times from the same patient, as well as the fact that each patient has a characteristic transcriptomic profile. Data show results from 5 patients of the randomized controlled trial with and without AlaGln supplementation (one patient yielded insufficient RNA under one condition and was therefore excluded from paired analysis).

Supplementary Figure 2

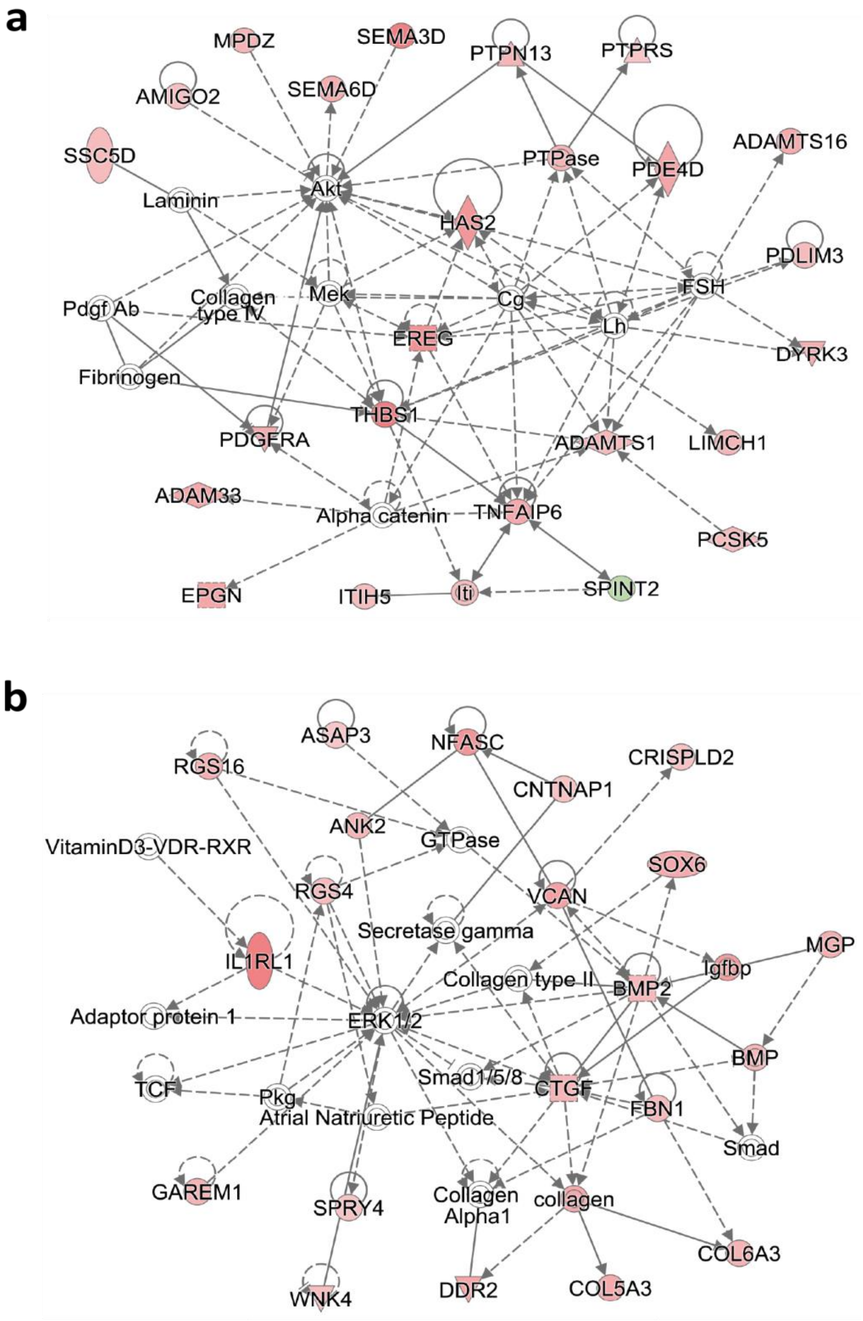

Top networks obtained from differentially abundant transcripts in peritoneal effluent cells. RNA-seq data was obtained from peritoneal effluent cells harvested from PET samples in the randomized clinical trial of PDF supplemented with or without AlaGln. Top-ranked (a) and third-ranked interaction networks (b) generated from differentially expressed genes. Each node represents a gene, and edges represent interaction(s) between or among genes. The node color indicates up-regulation (red) or down-regulation (green). (See Fig. 5b for second-ranked interaction network).

Supplementary Figure 3

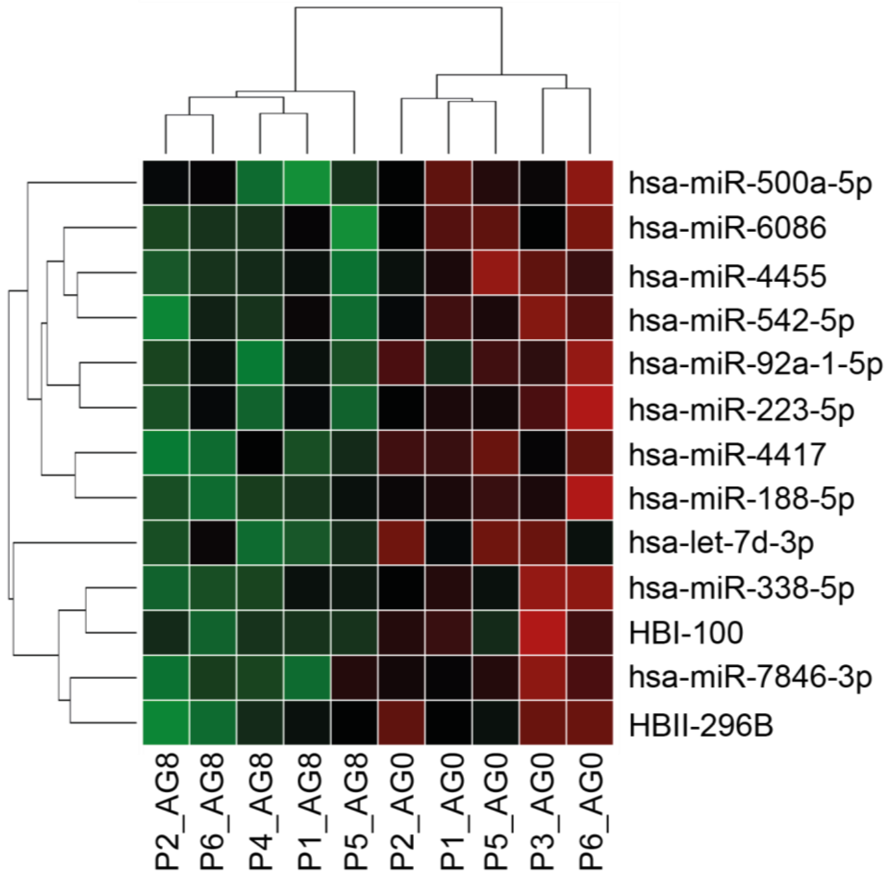

Heatmap of differentially abundant miRNAs. Heatmap rows depict differentially expressed genes, and columns depict indicated samples. Relative gene expression level is shown in pseudocolor from -3 to +3, with green representing down-regulation and red up-regulation. Data show results from 5 patients of the randomized controlled trial with and without AlaGln supplementation (one patient yielded insufficient RNA under one condition and was therefore excluded from paired analysis).

Supplementary Table 1: Blood parameters

Endpoint	PDF without AlaGln	PDF with 8 mM AlaGln	PDF with 16 mM AlaGln	*p*-value[a]	*p*-value[b]
Erythrocytes (T/l)	3.6 (3-4)	3.9 (3-4)	3.8 (4-5)	0.06	0.06
Haemoglobin (g/dl)	10.6 (9-12)	11.1 (10-12)	11.1 (11-13)	0.19	0.03
Leucocytes (G/l)	7.1 (6-9)	7.3 (6-10)	7.2 (5-8)	0.06	0.56
Platelets (G/L)	266 (220-387)	271 (232-376)	265 (239-378)	0.99	0.99
Neutrophil granulocytes (%)	70 (54-79)	74.5 (67-79)	69.5 (61-78)	0.13	0.88
Monocytes (%)	6.5 (5-11)	6.5 (2-8)	5.5 (5-10)	0.47	0.06
Lymphocytes (%)	21 (14-27)	18 (10-22)	18 (14-32)	0.13	0.88
Eosinophils (%)	2 (1-7)	3 (1-5)	1.5 (0-4)	0.99	0.38
Basophils (%)	1 (0-3)	1 (0-1)	1.5 (0-2)	0.75	0.99
Bicarbonate (mmol/L)	25.4 (23-27)	25.1 (23-28)	24.8 (23-26)	0.8	0.22
pH	7.4 (7-7)	7.3 (7-7)	7.3 (7-7)	0.44	0.13
Calcium (mmol/l)	2.3 (2-2)	2.3 (2-2)	2.3 (2-2)	0.06	0.56
Chloride (mmol/l)	94 (85-96)	94 (89-96)	94 (86-96)	0.75	0.99
Phosphorus (mmol/l)	1.2 (1-2)	1.6 (1-2)	1.6 (1-2)	0.22	0.44
Potassium (mmol/l)	4.4 (3-5)	4.6 (3-5)	4.4 (3-5)	0.44	0.84
Sodium (mmol/l)	136.5 (127-140)	137.5 (129-139)	135 (130-139)	0.44	0.63
Total protein (g/l)	57.8 (53-72)	56.1 (53-68)	59.3 (53-69)	0.31	0.99
Albumin (g/l)	37.5 (36-42)	37.4 (37-42)	40 (34-45)	0.25	0.31
Glucose (mg/dl)	100.5 (82-315)	96.5 (79-323)	107 (71-185)	0.99	0.22
Creatinine (mg/dl)	8.6 (6-15)	9.6 (7-18)	9.6 (6-17)	0.06	0.56
C-reactive protein (CRP) (mg/dl)	0.2 (0-1)	0.1 (0-1)	0.1 (0-1)	0.63	0.99
Urea nitrogen (BUN) (mg/dl)	59.5 (37-76)	67.8 (44-88)	62.8 (39-72)	0.03	0.84
Uric acid (mg/dl)	5.7 (5-8)	5.7 (5-7)	5.5 (5-7)	0.44	0.16
Lactic dehydrogenase (LDH) (U/L)	187 (137-298)	181.5 (154-206)	198.5 (172-233)	0.69	0.56
Cholesterol (mg/dl)	213.5 (143-260)	200.5 (114-263)	203 (139-267)	0.22	0.75
Alanine aminotransferase (ALT) (U/L)	18.5 (12-32)	13.5 (12-26)	15.5 (12-23)	0.22	0.25
Aspartate aminotransferase (AST) (U/L)	18 (14-25)	15 (9-28)	14.5 (10-27)	0.13	0.25
Gammaglutamyltransferase (GGT) (U/L)	17.5 (9-27)	16.5 (9-24)	17.5 (8-29)	0.38	0.75
High density lipoprotein cholesterol (mg/dl)	53 (39-91)	51.5 (32-102)	46.5 (34-79)	0.88	0.03
Low density lipoprotein cholesterol (mg/dl)	120.2 (71-166)	119.2 (55-142)	127.8 (82-163)	0.31	0.4
Triglycerides (mg/dl)	154.5 (98-282)	120 (89-284)	124 (108-305)	0.66	0.99
Alkaline phosphatase (U/L)	70 (45-126)	60 (1-130)	76 (45-136)	0.72	0.63
Bilirubin total (mg/dl)	0.3 (0-1)	0.3 (0-1)	0.3 (0-1)	0.69	0.44
CA-125 (kU/l)	26.6 (13-40)	26.9 (13-37)	25.3 (14-60)	0.99	0.44
Creatine Phosphokinase (U/L)	91 (78-407)	79 (61-120)	101.5 (70-128)	0.88	0.99
HbA1c (%)	5.4 (5-9)	5.8 (5-8)	5.9 (4-8)	0.63	0.99

Data are represented as median (range) of all 6 patients enrolled in the feasibility trial. Wilcoxon matched-signed rank test was used to calculate *p*-values. [a] comparison control without AlaGln versus 8 mM AlaGln in PDF. [b] comparison control without AlaGln versus 16 mM AlaGln in PDF. [c] blood parameters are at the 2h PET time point. Uncorrected *p*-values are shown.
